# Advancing sustainability science for the SDGs

**DOI:** 10.1007/s11625-018-0645-3

**Published:** 2018-11-14

**Authors:** Mark Stafford Smith, Christina Cook, Youba Sokona, Thomas Elmqvist, Kensuke Fukushi, Wendy Broadgate, Marcin Pawel Jarzebski

**Affiliations:** 1 CSIRO Land and Water, Canberra, Australia; 2 Montreal, Canada; 3 South Centre, Geneva, Switzerland; 4 Stockholm Resilience Centre, Stockholm University, Stockholm, Sweden; 5 Integrated Research System for Sustainability Science (IR3S), The University of Tokyo Institute for Advanced Study (UTIAS), The University of Tokyo, Tokyo, Japan; 6 Future Earth Secretariat Global Hub, ℅ Royal Swedish Academy of Sciences, Stockholm, Sweden

## Introduction

In September 2015, the world’s leaders agreed to the United Nations’ Agenda 2030 for sustainable development (UN 2015), including 17 sustainable development goals (SDGs). The SDGs provide a remarkable common global vision towards a safe, just and sustainable world for all human beings to thrive on the planet. The goals are seen as ambitions, and challenges, for all countries of all income levels, without exception. Between them, they provide a more resolved view of sustainable development than simply talking about economic, social and environmental dimensions; indeed, most goals are at least partially integrated across these dimensions. As such, the SDGs provide a normative framing that is “indivisible and universal” for the emerging discipline of sustainability science.

From the perspective of being “indivisible”, there is a growing appreciation of the interactions and dependencies among the goals, both in terms of substance and for coherent policy alignment. A key area that exemplifies the need to manage interactions relates to progress on climate change, the subject of SDG13 as well as the UN Framework Convention on Climate Change. The latest scientific findings on climate change show that the hoped-for plateauing of growth in greenhouse gas emissions has not yet occurred (Jackson et al. 2017), and global warming in 2017 reached 1 °C above pre-industrial conditions (WMO 2018). Research reaffirms that further warming will occur, which will cause coupled changes in all components of the climate system and amplify existing risks faced by natural and human systems (IPCC Working Group I 2013). Reversing this trend will require a major departure from business-as-usual and a change from incremental to exponential reductions in greenhouse gas emissions (e.g. Rockström et al. 2016). However, success on climate change will depend on aligning with other SDGs (e.g. SDG12 on responsible consumption and production); and success in meeting other SDGs will in turn depend on controlling climate change (e.g. hunger, SDG2). A key role of the research community is to provide knowledge to understand synergies and trade-offs so that challenging decisions can be made to maximise the synergies and minimise trade-offs (Griggs et al. 2014).

Although the SDGs are conceived as “universal”, that is, applying to all nations, it is also challenging that nations (including various levels of government, businesses and organisations) will need to take deeply differentiated and context-specific actions to achieve the objectives of Agenda 2030. Despite the need for global outcomes, most implementation will be local. For low-income countries, the main concerns are to bring national development objectives focused on all aspects of poverty alleviation together with issues of economic growth and the enhancement of human well-being. Meanwhile, higher income countries must recognise that they are often ‘under-developed’ in terms of SDG targets associated with issues such as reducing waste, obesity, greenhouse gas emissions and overall resource use. In these countries, the onus will be on delivering significant reductions in the impacts of resource use, nowhere better illustrated than with regard to reducing greenhouse gas emissions. Yet in both lower and higher income countries, the temptation will be to cherry-pick goals that are easily achieved.

The SDGs thus offer an opportunity to align the imperative of climate change action and sustainable development goals at local, national and global scales, at the same time defining key challenges for sustainability science in its support of policy. These challenges also resonate with the agenda of Future Earth, a major initiative on research and innovation for global sustainability, which aims to develop scientific knowledge and systemic solutions towards achieving both the SDGs and a healthy planet.

This Special Feature thus explores some aspects of how sustainability science can support the transformations needed to achieve sustainable development. The papers are based on presentations delivered at the 7th International Conference on Sustainability Science (‘ICSS 2017’) “Global Goals—New Approaches to Knowledge Generation—Challenges and Solutions from Local to Global Scales”, hosted by Future Earth, the University of Tokyo and the Stockholm Resilience Centre in August 2017. The conference brought together members of Future Earth’s emerging new Knowledge-Action Networks and Innovation Labs, both briefly described below. Accordingly, this editorial provides the background to these activities: the second section describes Future Earth’s Knowledge-Action Networks, the next section outlines the SDG labs, and then the following section introduces the remaining papers in the Special Feature and draws conclusions for both the progress of sustainability science as a discipline, and also the policy process around the SDGs.

## Future Earth and its Knowledge-Action Networks

The process of integrating knowledge—and approaches to creating knowledge—across different academic disciplines, epistemologies, and societal sectors generally happens in an ad hoc and incomplete manner. Indeed, in academia, efforts to integrate social sciences with natural sciences and humanities, and link the result with non-scientific knowledge are less rewarded or supported than detailed investigations within narrow disciplinary confines (e.g. Bromham et al. 2016; Haider et al. 2017; Rivera-Ferre et al. 2013). The challenge of diffuse and fragmented knowledge is pervasive in global environmental change research (e.g. see Future Earth 2013), and addressing this challenge is fundamental to delivering the ‘indivisibility’ of the SDGs. Future Earth was created to address this structural challenge, with a specific mandate to integrate across disciplines and to incorporate knowledge from beyond the bounds of academia to address the pressing problems created by global environmental change.

Since its inception in 2015, Future Earth has created Knowledge-Action Networks of people and organisations collaborating to build the knowledge and tools needed to tackle the greatest sustainability challenges of our time. Future Earth works to strengthen and expand these networks by (i) building communities and mobilising capacity to collaborate on research and innovation in each network’s scope (e.g. by hosting conferences and workshops, supporting fellowships, and facilitating strategic collaborations); and (ii) facilitating, co-designing and synthesising research to scale solutions across sectors and geographies (e.g. by seeding projects to catalyse transformations, highlighting priorities to funders, and co-disseminating new knowledge to drive action).

Future Earth recognises that networks alone will not overcome the more intransigent obstacles of knowledge fragmentation that partially led to its creation. Still, the development of this particular form of network aims to mobilise an emergent organisational innovation to address global issues. Future Earth also hosts a series of global research projects, networks of more disciplinary communities which have been driving global environmental change research for three decades. The newer Knowledge-Action Networks aim to leverage the fundamental science and knowledge produced by the Global Research Projects, and work toward Future Earth’s goal to “encourage co-design and co-production (…) by researchers in collaboration with various stakeholders in governments, industry and business, international organisations, and civil society” (Future Earth 2013).

Future Earth’s Knowledge-Action Networks (KANs) focus on themes such as health, cities and oceans. Integration across the KANs and the understanding of trade-offs and synergies between SDGs are tackled in an integrated way (cf. Nilssen et al. this issue) in the Future Earth work on science for Earth targets (for a full list of the networks, see http://www.futureearth.org). Several of the papers in this issue draw on the initial experiences of or contribute to these knowledge-action networks (e.g. Harms et al. this issue).

## SDG labs

The Social Innovation Lab concept, developed by the University of Waterloo for the Rockefeller Foundation (Westley et al. 2015), has been used around the world to catalyse change. Ahead of ICSS 2017, Future Earth used this approach to initiate a number of innovation labs focused on SDG implementation, under the title ‘SDG Labs’. The design was deliberately experimental but empowering, and participants were provided with a set of guidelines that Future Earth and the Stockholm Resilience Centre had developed from the Social Innovation Lab approach. The approach focused on stimulating local solutions across the globe towards meeting the SDGs. The labs brought together a range of researchers from different disciplines with other sectors of society to develop solutions to complex local problems. By focussing on prototype or scalable solutions that could lead to sustainability transformations, the Labs offered inspiration to the communities involved and others learning from them.

The outcomes of this initial set of 16 SDG labs were presented at ICSS 2017, and covered ideas such as integrated solutions for water in Indonesia (‘Water Warriors’), bringing indigenous knowledge into decision-making on health in the Pacific Islands, flooding and water issues as well as engaging youth in Africa, and to design how to map knowledge about the SDGs in Australia. The results of the SDG Labs are presented in a companion e-book (Springer, under development), where more details about the process may be found; one example is described in this Special Feature (Maher et al. 2018).

The SDG Lab process was unashamedly experimental within the framing of sustainability science, exploring the challenges of creating local solutions for sustainable development whilst considering how to scale towards more universal outcomes. The labs raised issues such as barriers to systemic change, power (im)balances, the selection of participants and partners, opportunism and timing, the importance of agents of change, and the need for ‘heartware’ to engage people; these issues are not new, but contribute to a growing set of context-sensitive case studies aimed at the SDGs. Many activities around the world are contributing to such experimentation and it is important that sustainability science continues to synthesise and learn from these efforts. In the Future Earth community, the Lab concept is being picked up across the networks as one process to stimulate collaboration between researchers and innovators on context-specific but scalable solutions. For example, SDG Labs were also developed in association with the *Seedbeds of Transformation Conference* held in South Africa in 2018 (http://www.seedbeds.futureearth.org). As such, the aim is to evolve a positive tool to help catalyse change and bring about momentum in transformations supported by research).

## Building on these issues

The principle objective of the ICSS 2017 is to feed into the High-Level Political Forum of the UN SDG process. The conference aims to create an output that showcases and maps the sustainability science of the Future Earth community—to support politicians charged with implementing the SDGs by giving them a navigation tool for using the relevant sustainability science. To accomplish this objective of the conference, eight sessions were organised. In this Special Feature, seven articles are included to reflect each session and discussion conducted on its theme.

Nilsson et al. (2018) in their paper explore the mapping of interactions between the sustainable development goals, drawing on “a major international research study applied to the SDGs on health, energy and the ocean, it analyses how interactions depend on key factors such as geographical context, resource endowments, time horizon and governance”. Nilsson et al. (2018) synthesise “experiences and insights from the application of a new conceptual framework for mapping and assessing SDG interactions using a defined typology and characterization approach”. Nilsson et al. (2018) examine “the future potential, barriers and opportunities for applying the approach in scientific research, in policy-making and in bridging the two through a global SDG Interactions Knowledge Platform as a key mechanism for assembling, systematising and aggregating knowledge on interactions”.

Lindgren et al. (2018) discuss sustainable food systems from the health perspective. The authors discuss “opportunities for and challenges to sustainable food systems from a human health perspective by making the case for avoiding the transition to unhealthy less sustainable diets (using India as an exemplar), reducing food waste by changing consumer behaviour (with examples from Japan), and using innovations and new technologies to reduce the environmental impact of healthy food production”. Their paper touches on “two of the challenges to achieving healthy sustainable diets for a global population, i.e. reduction on the yield and nutritional quality of crops (in particular vegetables and fruits) due to climate change; and trade-offs between food production and industrial crops”.

Martinez-Harms et al. (2018) discuss natural assets, and the concept and activities of the Natural Assets KAN. Their paper frames “Future Earth around natural assets emphasising the recognition of pluralism and identifying the challenges of translating different visions about the role of natural assets, including via policy formulation, for local to global sustainability challenges”. The discussion by Martinez-Harms et al. (2018) will be useful in developing inter- and transdisciplinary solutions for human–environmental problems.

Bengtsson et al. (2018) examine the transformation of systems of consumption and production for the purpose of achieving the SDGs. They show that “while the efficiency approach contains essential elements of a transition to sustainability, it is by itself highly unlikely to bring about sustainable outcomes. Concomitantly, research also finds that volumes of consumption and production are closely associated with environmental impacts, indicating a need to curtail these volumes in ways that safeguard social sustainability, which is unlikely to be possible without a restructuring of existing socioeconomic arrangements”. “Based on this determination, this paper provides some suggestions on how governments and other actors involved in SDGs operationalisation could more effectively pursue SCP from a systemic standpoint and use the transformation of systems of consumption and production as a lever for achieving multiple sustainability objectives”.

Elmqvist et al. (2018) discuss ‘urban tinkering’. They define tinkering as “a mode of operation, encompassing policy, planning and management processes, that seeks to transform the use of existing and design of new urban systems in ways that diversify their functions, anticipate new uses and enhance adaptability, to better meet the social, economic and ecological needs of cities under conditions of deep uncertainty about the future”. This approach has the potential to substantially complement and augment conventional urban development.

Maher et al. (2018) discuss design principles and opportunities for integrating design thinking with sustainability science towards achieving the SDGs. Maher et al. (2018) “examine the process of designing MetaMAP: an interactive graphic tool for collaborating to understand social–ecological systems and design well-integrated solutions. MetaMAP was created using Research through Design methods which integrate creative and scientific thinking. By applying design thinking, researchers and practitioners from different backgrounds undertook multiple cycles of problem framing, solution development, testing and reflection”.

Other key threads that emerged during ICSS 2017 included: meeting the SDGs in an integrated way (‘indivisible’), how approaches to this could be scaled up to transform society (‘universal’), and how all of this could be facilitated by the evolving, even maturing, discipline of sustainability science with the newly expanding suite of tools it has available, as exemplified in the Special Feature papers that follow.

Nilsson et al. open by documenting a formal approach to assessing interactions among SDGs that argues for a systematic way of collating and quantifying benefits in this regard, following the reality that no individual SDG can be met without meeting all the others (noting that the balance and priorities may be very specific to individual countries). There follow a series of papers exploring issues of integration, transformation and scaling in more-or-less specific areas of the SDGs. Lindgren et al. emphasise links among targets for health and food. Harms et al. discuss the need to manage natural assets within a pluralist vision that recognises the importance of collaboration, equity and power. While the Sustainable Consumption and Production aspects of the SDGs identify a potentially transformative agenda, Bengtsson et al. show that the SDG targets are not generally expressed in the system-wide way that is needed to ensure global outcomes. Then, Elmqvist et al. explore tinkering as a tool that is sensitive to conditions of uncertainty and complexity in urban sustainability. Maher et al. show that bringing tools such as design thinking into sustainability research may assist the transdisciplinarity which is needed.

Van der Leeuw (2018) rounds off the collection by reflecting on these indicators of the revitalisation and maturing of sustainability science, whilst highlighting some challenges—our need to focus on a society subject to permanent change, understanding and stimulating innovation and change beyond a stability-focused paradigm, emphasising relationships as much as entities, rethinking the “idea of progress” in western cultures, and achieving modesty in the (still important but changing) role of research in society.

Overall, ICSS 2017 and this Special Feature show how sustainability researchers now need to:

- Enrich their theory and practice of deep interdisciplinarity and transdisciplinarity, to support, the indivisibility and universality of sustainable development as framed by the SDGs
- Emphasise the understanding of how to ‘tinker’ locally with an eye on how context-specific solutions may be used in other parts of the world
- Support solutions and scaling by encouraging and documenting an explosion of experiments involving researchers and stakeholders such as the SDG Labs within a context of broader networks
- Consider and where appropriate integrate different types of knowledge—indigenous, practitioner, policy, academic—and create tools that help practitioners to benefit equitably from that knowledge
- Recognise reflexively that “sustainability is itself an open-ended social learning process”, in the words of Francesca Farioli at the conference.

At the same time, key messages emerged for policy-makers:

- The indivisible intent of the SDGs requires a continued emphasis on maintaining coherence among policy areas at all levels of governance, in the face of the reality that many institutional incentives promote fragmentation: a powerful area for the practical expression of this principle is in ensuring the close alignment between work on climate change and the rest of the SDGs
- Ensuring the universality of Agenda 2030 requires a balanced focus on locally and nationally appropriate action, whilst understanding how this contributes the global outcomes that affect us all; allowing either side of the balance to dominate will undermine global sustainability and human well-being.
- These priorities can be brought together in practice by endorsing and encouraging many local action-learning experiments that link benefits across many SDGs, and which are actively networked and tracked to enable global learning
